# Influence of Light Quality on the Initial Development in Edible Brown Alga *Cladosiphon okamuranus*

**DOI:** 10.3390/plants15060895

**Published:** 2026-03-13

**Authors:** Yoichi Sato, Eri Inomata, Hikari Nagoe, Yuuichiro Numata, Atsuko Tanaka

**Affiliations:** 1 Bio-Resources Business Development Division, Riken Food Co., Ltd., Tagajo-shi 985-0844, Miyagi, Japan; 2 Nishina Center for Accelerator-Based Science, RIKEN, Wako 351-0198, Saitama, Japan; 3 Faculty of Science, University of the Ryukyus, Nishihara 903-0213, Okinawa, Japan

**Keywords:** *Cladosiphon okamuranus*, light wavelength, blue light, aquaculture, germination induction, nutrient

## Abstract

The impact of light quality on pre-germling formation was studied in the edible macroalga *Cladosiphon okamuranus*, cultivated in subtropical Japan. While the conditions for germination remain unclear, successful cultivation typically occurs in deeper nursery sites where light quality might play a crucial role. Initially, we compared light wavelengths at various depths, revealing that blue light (400–500 nm) predominated at approximately 7.5 m, where aquaculture nets were used for germination. Two experiments were conducted using discoid thalli. In the first, thalli were grown under four light conditions (white, blue, red, and a combination of blue and red) in sterilized seawater or nutrient-enriched PESI medium. Blue light resulted in the highest pre-germling formation rates in sterilized seawater, and notable rates were observed in the PESI medium across all wavelengths except red. The second experiment involved culturing thalli in sterilized seawater under white, blue, and red light at varying intensities (25 or 100 µmol photons m^−2^ s^−1^). The consistent promotion of pre-germling formation was observed with blue light, whereas red light produced no effects. These findings highlight the importance of blue light for germination in *C. okamuranus*, which can aid in optimizing nursery site and incubation conditions.

## 1. Introduction

In the marine ecosystem, sunlight is filtered through seawater, drastically cutting out red light (>600 nm) in the first 5 m from the surface, such that blue light (around 480 nm) predominates below 5 m and overall light intensity is cut by half at an approximately 15 m depth [1,2]. It is known that macroalgae utilize blue light; for instance, it induces gametogenesis, gametophyte growth and maturation, and early sporophyte development in brown algae [3,4,5,6,7], and also gamete release from gametangia in green algae [8]. Furthermore, the blue light regulates the ratio of the light-harvesting complex LHCII to its reaction center RCII in microalga *Chlamydomonas reinhardtii* [9]. Macro- and microalgae possess various blue light receptors, rhodopsins, phototropins, BLUF (blue-light sensors using flavin-adenine dinucleotide) proteins, and aureochromes, although those physiological functions are not entirely understood [10,11,12]. In land plants, light wavelength is one of the most important stimuli for the induction of germination; however, there is less information about the influence of particular light wavelengths on algal germination.

The light condition is one of the factors most controllable by cultivation depth in actual seaweed cultivation sites. In fact, cultivation depth is regulated to avoid discoloring in *Undaria pinnatifida* [13] and photoinhibition in *Kjellmaniella crassifolia* [14,15]. However, depth changes both the quality and quantity of light in the water. A detailed study about the effects of light quality and quantity on macroalgae is therefore crucial for improving cultivation procedures.

*Cladosiphon okamuranus* Tokida, 1942 (Class Phaeophyceae) is a subtropical edible brown alga [16,17]. In Japan, cultivation techniques for this species were developed in Okinawa and Kagoshima Prefectures in 1972 [18,19,20], and its industrial cultivation began in Okinawa Prefecture in 1977 [21]. In the past 50 years, this species has become a widely available food item in Japan. For local communities in Okinawa Prefecture, *C. okamuranus* is one of the most important cultivated species (locally known as ‘Okinawa mozuku’). However, there has been a marked fluctuation in its annual yield, with values ranging from 8000 to 25,000 metric tons (for the years 2006–2021 [22]).

Cultivation of *C. okamuranus* differs from that of other macroalgae in that cultivation nets are moved to different depths at different growth stages to maximize yield (Figure S1) [20,23,24,25]. The cultivation net is moved to the nursery site after approximately 4 weeks of cultivation in the land-based tank, during which the spores attach to the net and discoid thalli form. This nursery site is located in a relatively deep area, and its suitability is assessed using empirical criteria. Typically, the net is placed at the nursery site for 15–20 days to germinate erect thalli and then moved to a post-nursery location for 35–40 days to promote further thallus growth. Although the exact number of days varies somewhat by location, moving the nets before the main farming period is generally practiced. The post-nursery is located in a shallower area than the nursery site with higher light intensity, which promotes rapid thallus elongation [20]. Finally, the cultivation nets are placed in the farming area, which is located at a deeper site than the post-nursery site to avoid storm damage. Local culture practitioners have identified some nursery sites as either ‘good’ or ‘poor’ [23], apparently based on nutrient conditions [20,23] and current condition [26]. It is also known that initial thallus development is affected by several other factors, including water depth [26], irradiance, and nutrient concentrations [27]. However, the optimal conditions for promoting the initial development of thallus remain unclear. An investigation is also necessary to understand the effects of various environmental factors, including light conditions at the nursery site.

In *C. okamuranus*, the effects of light on germling and growth remain largely unknown. To provide preliminary insights into this subject, the present study explores the initial development of erect thalli of *C. okamuranus* under blue (B), red (R), and white (RGB) light. Initially, ambient light conditions at commercial aquaculture sites for this alga were measured to determine the irradiance spectra at nursery, post-nursery, and farming locations. Based on these measurements, two experiments were conducted: (1) to examine the effects of different wavelengths under varying nutrient conditions, and (2) to study the effects of light intensity. The aim was to identify ways to enhance cultivation techniques for this species.

## 2. Results

### 2.1. Field Survey of the Irradiance Spectra at the Aquaculture Sites in Okinawa Prefecture

**Figure 1:**
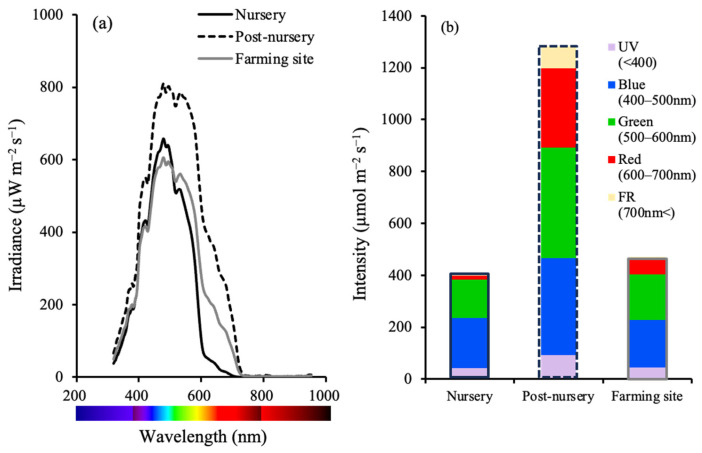
The wavelength spectra on the sea bottom at three aquaculture sites (nursery, post-nursery, and farming site) for Chinen, Okinawa Prefecture (**a**), and the light intensity ratio for UV (<400 nm), blue (400–500 nm), green (500–600 nm), red (600–700 nm), and far-red (700 nm<) ranges at sites (**b**).

The irradiance spectra on the sea bottom at three aquaculture sites (nursery at 7.5 m, post-nursery at 2 m, and farming site at 3 m) for Chinen, the southern part of Okinawa Island, are shown in Figure 1. At Chinen, water depth varied among the aquaculture sites, with the nursery located in the deepest water. Light intensity was the lowest at the deepest nursery, increasing successively at the shallower farming site and post-nursery (Figure 1a). Regarding wavelength spectra, the UV (<400 nm) and blue (400–500 nm) ranges remained nearly constant across all three locations (Figure 1a,b). Meanwhile, the nursery and farming site showed lower values than the post-nursery in the blue and green ranges (400–600 nm), and were remarkably low in the red (600–700 nm) and above far-red ranges (700 nm<) in the nursery (Figure 1a,b).

### 2.2. Experiment 1: Effects of Light Wavelength Combinations on Promoting Pre-Germling Formation Under Two Different Nutrient Conditions

**Figure 2:**
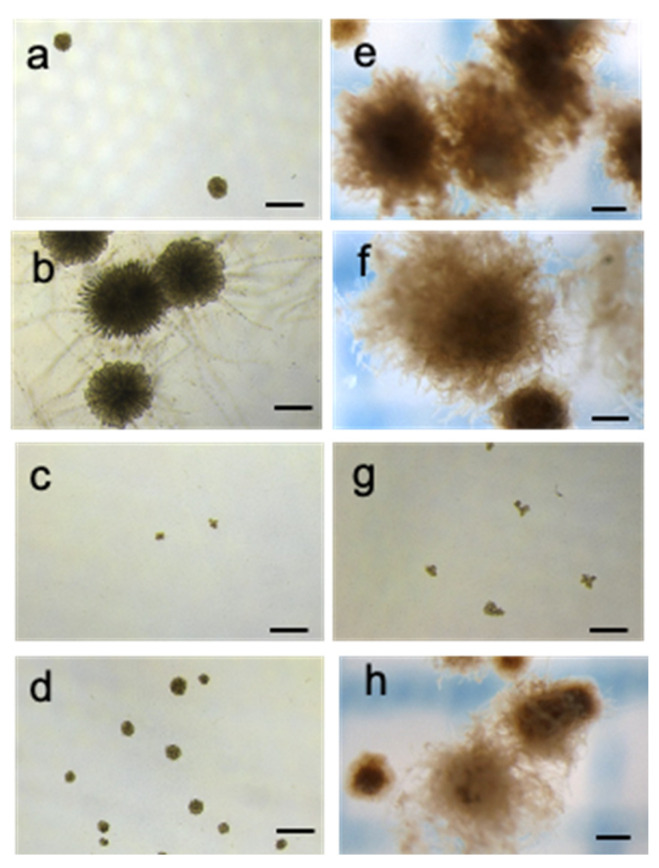
Images of *Cladosiphon okamuranus* discoid thalli were captured in sterilized seawater without nutrient enrichment (left, panels (**a**–**d**)) and in the PESI medium (right, panels (**e**–**h**)) at different light wavelengths on Day 42 of the experiment. Panels (**a**,**e**) show white light (RGB), panels (**b**,**f**) show blue light (B), panels (**c**,**g**) show red light (R), and panels (**d**,**h**) display a combination of blue and red light (RB). The light intensity was maintained at 50 µmol photons m^−2^ s^−1^, with the RGB and RB components adjusted to ensure equal light intensity across the R, green (G), and B wavelength ranges. Specifically, each of the R, G, and B components of RGB was set to approximately 17 µmol photons m^−2^ s^−1^, while the R and B components of RB were set to 25 µmol photons m^−2^ s^−1^. Scale bars indicate 200 μm.

We defined the discoid thallus with an obviously erected portion as pre-germinating. Figure 2 shows images of *C. okamuranus* discoid thalli in sterilized seawater or the PESI medium under one of four different wavelength combinations at Day 42. Discoid thalli under RGB and B formed pre-germlings in sterilized seawater (Figure 2a,b). In the PESI medium, forming pre-germlings was detected at all wavelengths except R in the PESI medium (Figure 2e,f,h). Under R and blue-red (RB) in sterilized seawater and R in the PESI medium, pre-germlings did not form, and discoid thalli did not increase in size (Figure 2c,d,g).

**Figure 3:**
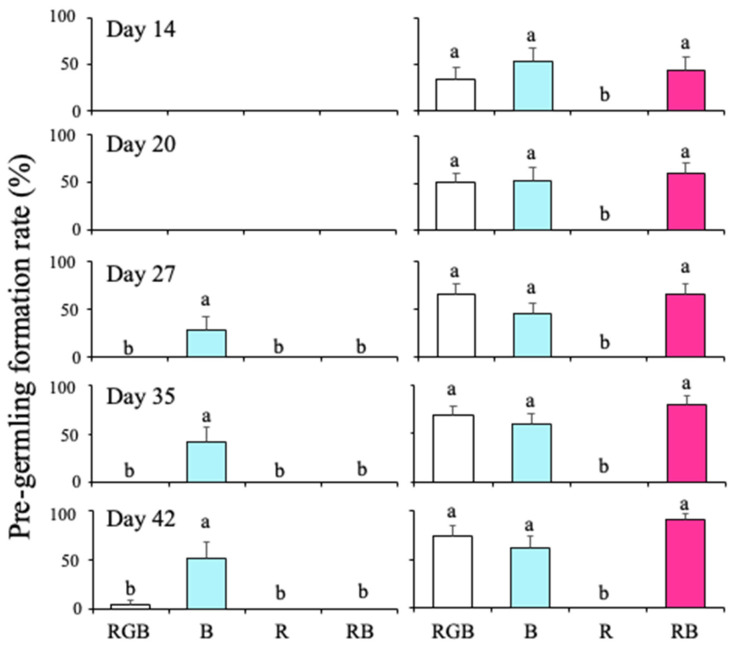
The pre-germling formation rate of *Cladosiphon okamuranus* was measured in sterilized seawater (**left**) and the PESI medium (**right**) at various light wavelengths over different incubation days. The light intensity was set at 50 µmol photons m^2^ s^−1^, and the RGB and RB components were adjusted to ensure consistent light intensity across each wavelength range. Specifically, each of the R, G, and B components of RGB was set to approximately 17 µmol photons m^−2^ s^−1^, while the R and B components of RB were set to 25 µmol photons m^−2^ s^−1^. Error bars represent the standard error of the pre-germling formation rate, and letters (a and b) indicate statistical differences between the light wavelengths.

Pre-germling formation rates in sterilized seawater and in the PESI medium at different wavelengths of light are shown in Figure 3. At all measurement dates, the pre-germling formation rate was significantly affected by nutrient condition and wavelength (Table S1). Moreover, there was an interaction between nutrient condition and wavelength (Table S1). In sterilized seawater, discoid thalli did not form pre-germlings until Day 20 across all wavelengths (Figure 2). Under B, pre-germlings were detected from Day 27 (Figure 2). At Day 42, although the pre-germling formation was found under RGB, the formation rate under B was 52.0%, significantly higher than that under RGB (*p* < 0.01, Figure 3). Under R and RB conditions, pre-germlings were not detected throughout the cultivation period.

In the PESI medium, pre-germlings had already formed at Day 14 under all wavelengths except R (Figure 3), and the formation rates were not significantly different among RGB, B, and RB. Under R, pre-germlings were not detected throughout the cultivation period, as in the sterilized seawater condition.

### 2.3. Experiment 2: Effects of Light Intensity on Promoting Pre-Germling Formation at Different Light Wavelengths

**Figure 4:**
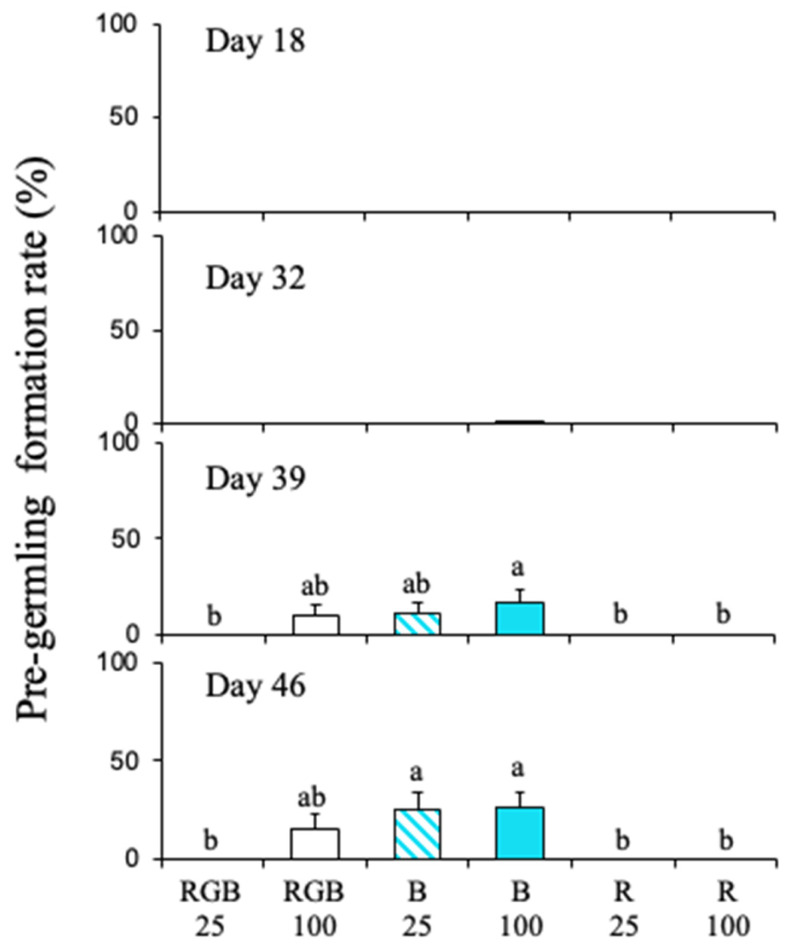
Bar charts presenting the pre-germling formation rate of *Cladosiphon okamuranus* in sterilized seawater, at various wavelengths, at light intensities of 25 or 100 µmol photons m^−2^ s^−1^, measured over four different days. The labels at the bottom of the charts indicate the wavelength ranges (coded as RGB, B, and R), with the corresponding light intensities provided below. The RGB light intensity was adjusted to ensure equal values across each wavelength range. Specifically, each of the R, G, and B components of RGB was set to approximately 8 µmol photons m^−2^ s^−1^ for 25 µmol photons m^−2^ s^−1^ and 33 µmol photons m^−2^ s^−1^ for 100 µmol photons m^−2^ s^−1^. Error bars represent the standard error of the pre-germling formation rate, and letters (a, b, ab) indicate statistical differences between the effects of light intensity and wavelengths.

Pre-germling formation rates in sterilized seawater under three different light wavelengths are shown in Figure 4. Until Day 18, discoid thalli did not form pre-germlings under any conditions. After Day 39, pre-germling formation rate was significantly affected by both light intensity and wavelength (Figure 4; Table S2). However, there was no interaction between light intensity and wavelength throughout the experiment. As in Experiment 1, no pre-germlings were observed under R, and the same result was found for all light intensities tested (Figure 4). Pre-germlings were formed only under B at 100 µmol photons m^−2^ s^−1^ at Day 32 (Figure 4). At Day 39, pre-germlings were formed under B at both light intensities and under RGB at 100 µmol photons m^−2^ s^−1^ (Figure 4). At Day 46, pre-germling formation rates under B at both light intensities were significantly higher than those under R at both light intensities and RGB at 25 µmol photons m^−2^ s^−1^ (*p* < 0.05; Figure 4).

## 3. Discussion

The influence of environmental factors on the morphogenesis of *Cladosiphon okamuranus* can be summarized as follows (Table S3): (1) Erect sporophyte formation occurs at 15–25 °C [28,29,30]. (2) Pre-germling formation is promoted under nutrient-enriched conditions, regardless of light intensity. However, under nutrient-free conditions, high light intensity (80 µmol photons m^−2^ s^−1^) is required for this process [27]. (3) Regarding microthallus dimorphism of this alga, the sporophyte-component morphotypes were also observed to be most abundant at 15–25 °C [31]. Despite these observations, none of these studies has yet identified the key factors in morphogenesis. The present study revealed that the pre-germling formation rate of *C. okamuranus* varies significantly across different wavelengths of light. Although the effect of light wavelength was more pronounced without nutrient enrichment, similar trends were observed under enriched nutrient conditions. In particular, B markedly promoted pre-germling formation across nutrient conditions and light intensities, acting as a key factor. For Phaeophyceae, to which *C. okamuranus* belongs, although there have been many studies concerning the effects of blue light on maturation [3,4,6], this study is the first report on the germination promotion of blue light. While red/far-red light is generally known to affect germination in terrestrial plants, the fact that brown algae are induced to germinate by blue light is a reasonable consequence consistent with the light environment of their habitat (Figure 1). As a blue-light receptor, the brown algae have aureochromes, which are known to be involved in morphogenesis [12]. In the genome of *C. okamuranus*, there are homologs of aureochromes identified in the genome of *Ectocarpus siliculosus* [32,33], and *C. okamuranus* possibly detects the blue light with this photoreceptor. This speculation would become evidence through further studies of gene expression of aureochromes homologs across treatments, inhibitor experiments, or photoreceptor action-spectrum proxies.

The nursery is located at a site deeper than the post-nursery and farming sites at Chinen because fishermen empirically believe that erect thallus germination is promoted at deeper locations (Hayashi, personal communication). In the nursery, the proportion of B was high, while the proportions of R and FR were markedly low (Figure 1). The finding that blue light promoted the formation of pre-germlings in sterilized seawater may be duplicating the wavelength and nutrient conditions at the nursery site. Surveying irradiance spectra should be an effective method for selecting an optimal nursery site, and future management methods should be based on satellite imagery. The present study strongly supports the conclusion that depth control at the nursery stage was synonymous with light-quality control in the field. However, underwater irradiance spectra will vary with season, weather, and solar radiation, so regular collection of irradiance spectra data at aquaculture sites for *C. okamuranus* will be necessary to inform best practice.

Research on the light conditions necessary for sporophyte formation in *Scytosiphon lomentaria*, which is in the same order (Ectocarpales) as *C. okamuranus*, indicates that short-day conditions promote sporophyte development. Although specific optimal irradiance values were not defined, low irradiance levels have been found to be adequate for sporophyte growth (Table S3) [34,35]. In the case of *C. okamuranus*, the formation of erect sporophyte thalli is boosted at moderate irradiance levels (1000–3000 lux) but is inhibited at higher levels (6000 lux) [28]. Additionally, the pre-germling formation of *C. okamuranus* is more significantly influenced by nutrient availability than by light intensity (Table S3) [27]. In this study, the effects of light intensity on pre-germling formation under non-enriched conditions were not observed under B, but they were apparent under RGB. Specifically, pre-germling formation was not observed at RGB 25 µmol photons m^−2^ s^−1^, whereas it was observed at RGB 100 µmol photons m^−2^ s^−1^. Considering that the amount of B at RGB 25 µmol photons m^−2^ s^−1^ is approximately 8 µmol photons m^−2^ s^−1^, it suggests there is a threshold for pre-germling formation in this alga between 8 and 25 µmol photons m^−2^ s^−1^. This finding implies that light intensity has a minimal effect on the morphogenesis of this species, indicating that blue light may act as a trigger for pre-germling formation.

Pre-germlings were formed at all wavelengths of light except R when nutrient supplementation was provided using the PESI medium. This finding is potentially useful for developing an incubation system using land-based tanks under controlled nutrient and light-wavelength conditions. Additionally, it has been observed that pre-germling formation in this alga was enhanced at an irradiance of 80 µmol photons m^−2^ s^−1^ when grown in sterilized seawater; however, with nutrient supplementation, pre-germling formation occurred even at a lower intensity of 15 µmol photons m^−2^ s^−1^ [27]. To promote the germination of erect thalli, using LEDs and outdoor tanks equipped with blue-selective filters, along with appropriate shading materials during nutrient supplementation, could be beneficial. However, there is a risk of contamination from diatoms and other algae under enriched conditions. To develop these techniques on an industrial scale, further studies are needed to determine the appropriate range of nutrient levels. Specifically, determining the optimal nutrient composition of Okinawan seawater—which this study did not address—will be crucial for long-term success. While such artificial methods may incur costs, they may ultimately prove to be cost-effective compared to relying on the unpredictable natural environment at the nursery. Selecting filtering materials that restrict all light except blue light from natural sources will amplify these cost benefits, creating a more sustainable and productive approach.

The cultivation of *C. okamuranus* is unique because the transfer of culture nets is widely practiced. In recent years, a similar cultivation technique has been proposed: submerging *Macrocystis* kelp in nutrient-rich deep water at night and bringing it to shallower, sunlit depths during the day [36]. In the current situation, in which the aquaculture environment is fluctuating greatly and poor growth and color fading of cultured seaweed are becoming more common [37,38], such proactive improvement techniques for successful aquaculture are likely to become increasingly important. The findings here suggest that consideration of underwater wavelength spectra could be a key factor in optimizing conditions at aquaculture sites. Further research is required to find the optimum conditions for the development of erect thalli of *C. okamuranus*. In addition, research focused on the morphology and biological characteristics of this species is needed to help stabilize aquaculture production.

## 4. Materials and Methods

### 4.1. Sample Collection and Stock Maintenance

**Figure 5:**
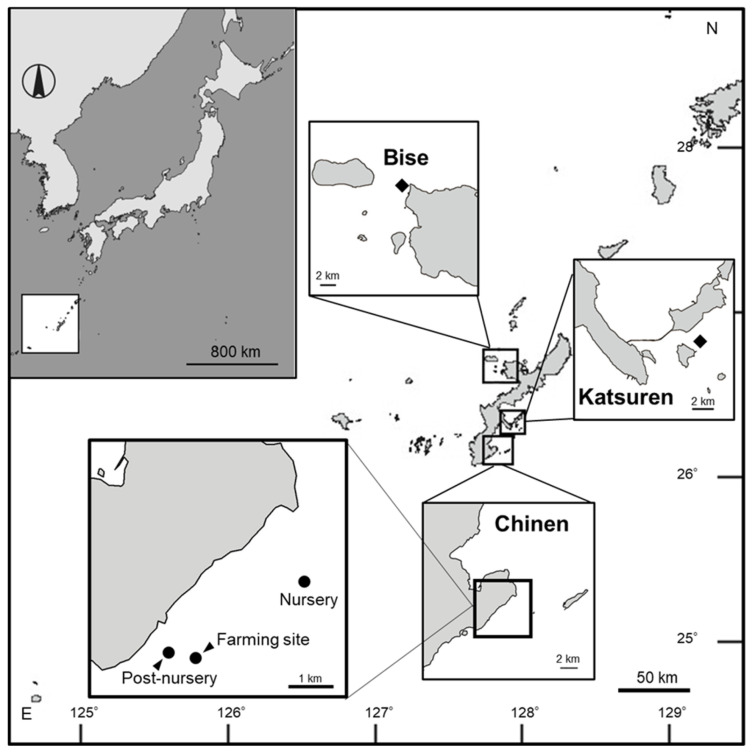
Map of the study sites. The rhombuses indicate the sampling sites of *Cladosiphon okamuranus* sporophytes for Experiments 1 and 2. The circles indicate the measuring points of the field survey.

Sporophytes of *C. okamuranus* cultivated in Okinawa Prefecture at Cape Bise (26°42′30″ N, 120°52′42″ E) and near the Katsuren Peninsula (26°19′48″ N, 127°57′44″ E) were collected at the beginning of April 2018 (Figure 5). Epiphytes growing on the sporophytes were removed, and the thalli were carefully washed with sterilized seawater. The sporophytes were transported by air within 3 days to the Algal Research Innovation Centre of Riken Food Co., Ltd. in Natori, Miyagi Prefecture. Sporophyte fragments of approximately 5 mm in total length were cultured in 1 L flasks containing sterilized seawater at room temperature (19–21 °C) under approximately 30 µmol photons of m^−2^ s^−1^ white LEDs (400–780 nm) during daytime. With a light microscope, plurilocular sporangia were observed forming at the tip of the sporophyte assimilatory filaments. A sterilized glass slide was placed on the bottom of the flask to obtain discoid thalli following the settlement of zoospores formed from the plurilocular sporangia. These discoids were then scraped from the glass slide and cultured in 6-well microplates, with one thallus per well, under 30–40 μmol photons m^−2^ s^−1^ fluorescent light (400–780 nm) for 12 h of light and 12 h of dark at 24 °C. For approximately one month, the discoid thalli were incubated until plurilocular sporangia were observed forming on the assimilatory filaments. These discoid thalli were then removed and placed into new microplate wells, and new discoid thalli were germinated from the zoospores. These cultures were repeated until it was possible to routinely prepare discoid thalli with plurilocular sporangia for the following experiments.

### 4.2. Field Survey of Light Wavelength Characteristics at the Two Culture Sites in Okinawa

A field survey was conducted at Chinen, the southern part of Okinawa Island, in October 2019 (Figure 5). Three different aquaculture sites were investigated: the nursery (26°08′57″ N, 127°50′18″ E), the post-nursery (26°08′06″ N, 127°47′53″ E), and the main farming site (26°08′20″ N, 127°48′55″ E). We used a TriOS RAMSES apparatus (TriOS Mess- und Datentechnik GmbH, Rastede, Germany) to measure the wavelength characteristics of undersea light. The sensor was fixed to the end of a fishing line, with the light receiver pointing upward. It was lowered until it touched the seabed, then gradually raised by reeling in the line to measure irradiance across the wavelength range 319–952 nm at different depths at each site.

The irradiance data were converted to light intensity (µmol photons m^−2^ s^−1^) by multiplying by a factor of 0.012 after comparing the data obtained with TriOS RAMSES and the LA-105 light sensor (Nippon Medical & Chemical Instruments, Osaka, Japan). The wavelengths were divided into 5 bins, namely Ultra Violet (UV; <400 nm), blue (400–500 nm), green (500–600 nm), red (600–700 nm), and far red (FR; >700 nm), and the proportion of each wavelength bin was calculated for each site.

### 4.3. Experiment 1: Effects of Light Wavelength Combinations on Pre-Germling Formation with or Without Nutrient Supplementation

Culture experiments were conducted from 15 June to 27 July 2018. On Day 0 (15 June), a total of 210 large, well-grown discoid thalli were selected, removed from their microplate well, and placed in seven 6-well microplates with five individuals per well. Three wells of each microplate were filled with sterilized seawater, and the other three with the PESI medium [39]. The medium in each well was replaced with fresh medium once per week throughout the experiments. The microplates were incubated (CN-40A Incubator, Mitsubishi Electric Engineering, Tokyo, Japan) at 24 °C [27] with illumination by LED light within the incubators (3LH-64; Nippon Medical & Chemical Instruments, Osaka, Japan) set at one of 4 different light wavelength combinations (Figure S1): blue (B), red (R), white (RGB), and red blue (RB). The wavelengths of B and R were set at 400–500 nm and 600–700 nm, respectively, as measured with a light analyzer (LA-105). The irradiance of RGB and RB was adjusted so that their values within their respective wavelength ranges were nearly equal. Each microplate was exposed to one of these 4 different wavelength conditions at a total intensity of 50 µmol photons m^−2^ s^−1^. Specifically, each of the R, green (G), and B components of RGB was set to approximately 17 µmol photons m^−2^ s^−1^, while the R and B components of RB were set to 25 µmol photons m^−2^ s^−1^. All incubations used a 12 h light:12 h dark photoperiod. Seven days after the start of the experiment, following the settlement of some zoospores from the plurilocular sporangia on the bottom of the microplate, the discoid thalli were removed from each well. From Day 14 (29 June), three images of each microplate well (total 9 images of each treatment, *n* = 9) were taken weekly using an inverted microscope (TS100, Nikon, Tokyo, Japan) or a stereomicroscope (SZX7, Olympus, Tokyo, Japan). The total number of discoid thalli and thalli with a “pre-germling” was counted and expressed per 4 mm^2^. The pre-germling indicates the initial development of an erect thallus and is defined as an aggregation of assimilatory filaments (pseudoparenchymatous tissue) at the center of the discoid thallus [27,28]. The pre-germling formation rate is expressed as the number of discoid thalli with a pre-germling as a percentage of the total number of thalli.

### 4.4. Experiment 2: Effects of Light Intensity on Promoting Pre-Germling Formation at Different Light Wavelengths

This experiment was conducted from 25 January to 12 March 2019. On 25 January (Day 0), eight 6-well microplates, each containing five discoid thalli from Katsuren, were prepared using the same method as used in Experiment 1. All wells were filled with sterilized seawater to avoid nutrient effects on the wavelength response. These microplates were placed in an incubator (CN-40A) at 24 °C. Illumination in the incubators (3LH-64) was set to one of 3 light wavelengths: B, R, or RGB. Each light wavelength was set at two light intensities: 25 µmol photons m^−2^ s^−1^ and 100 µmol photons m^−2^ s^−1^. Specifically, each of the R, G, and B components of RGB was set to approximately 8 µmol photons m^−2^ s^−1^ for 25 µmol photons m^−2^ s^−1^ and 33 µmol photons m^−2^ s^−1^ for 100 µmol photons m^−2^ s^−1^. Photoperiod was 12 h light and 12 h dark. From Day 18 (12 February), three images were taken of each microplate well (total 18 images of each treatment, *n* = 18) using an inverted microscope (TS-100) once a week, and the pre-germling formation rate was calculated as performed in Experiment 1.

### 4.5. Statistical Analysis

The statistical significance of differences in pre-germling formation rates was assessed using ANOVA. The effects of nutrient condition and light wavelength on the pre-germling formation rate in Experiment 1 and the effects of light intensity and wavelength on the pre-germling formation rate in Experiment 2 were determined by two-way ANOVA. Tukey’s multiple comparison test was performed as a post hoc test. These analyses were conducted after log transformation since some data were not normally distributed (Shapiro–Wilk test, *p* < 0.05) and did not show homogeneous variances (Levene test, *p* < 0.05). Analysis was conducted by JMP 14 (SAS Institute Inc., Cary, NC, USA).

## Data Availability

Data are contained within the article and Supplementary Material.

## Supplementary Materials

The following supporting information can be downloaded at https://www.mdpi.com/article/10.3390/plants15060895/s1. Table S1: Results of two-way ANOVA for pre-germling formation rate of *Cladosiphon okamuranus* in sterilized seawater or the PESI medium at different light wavelengths. Table S2: Results of two-way ANOVA for pre-germling formation rate of *C. okamuranus* in sterilized seawater at different light wavelengths at intensities of 25 or 100 µmol photons m^−2^ s^−1^. Table S3: The influence of environmental factors on the morphological development from discoid thallus to erect sporophyte of *Cladosiphon okamuranus* and *Scytosiphon lomentaria*. Figure S1: Illustration of the life cycle of *Cladosiphon okamuranus* (left) alongside photographs of the aquaculture process corresponding to each life stage of this species (right). a: Cultivation nets in a land-based tank for attaching spores and incubating discoid thalli; b: Nursery and post-nursery site at the sea bottom for germinating erect sporophytes from discoid thalli; c: Farming area for growing sporophytes until harvest. The life cycle illustration is based on Figure 1 from [31], which is a modified version of Figure 10 from [40]. Copyright for photograph c belongs to Mr. Akihisa Hayashi. Figure S2: Composition of LED lights set at 4 different light wavelength combinations measured by using a light analyzer (LA-105, Nippon Medical & Chemical Instruments, Osaka, Japan).

## References

[1] Ivanoff A., Waterman T.H. (1958). Factors, mainly depth and wavelength, affecting the degree of underwater light polarization. J. Mar. Res..

[2] Ivanoff A., Jerlov N., Waterman T.H. (1961). A comparative study of irradiance, beam transmittance and scattering in the sea near Bermuda. Limnol. Oceanogr..

[3] Lüning K., Dring M.J. (1972). Reproduction induced by blue light in female gametophytes of *Laminaria saccharina*. Planta.

[4] Lüning K., Dring M.J. (1975). Reproduction, growth and photosynthesis of gametophytes of *Laminaria saccharina* grown in blue and red light. Mar. Biol..

[5] Forster R.M., Dring M.J. (1994). Influence of blue light on the photosynthetic capacity of marine plants from different taxonomic, ecological and morphological groups. Eur. J. Phycol..

[6] Sato Y., Endo H., Oikawa H., Kanematsu K., Naka H., Mogamiya M., Kawano S., Kazama Y. (2020). Sexual difference in the optimum environmental conditions for growth and maturation of the brown alga *Undaria pinnatifida* in the gametophyte stage. Genes.

[7] Wang W.J., Sun X.T., Wang F.J. (2010). Effect of blue light on early sporophyte development of *Saccharina japonica* (Phaeophyta). Mar. Biol..

[8] Mine I., Okuda K., Tatewaki M. (1996). Gamete discharge by *Bryopsis plumosa* (Codiales, Chlorophyta) induced by blue and UV-A light. Phycol. Res..

[9] Shepherd H.S., Ledoigt G., Howell S.H. (1983). Regulation of light harvesting chlorophyll binding protein (LHCP) mRNA accumulation during the cell cycle in *Chlamydomonas reinhardtii*. Cell.

[10] Kianianmomeni A., Hallmann A. (2014). Algal photoreceptors: In vivo functions and potential applications. Planta.

[11] Ziegler T., Möglich A. (2015). Photoreceptor engineering. Front. Mol. Biosci..

[12] Takahashi F., Yamagata D., Ishikawa M., Fukamatsu Y., Ogura Y., Kasahara M., Kiyosue T., Kikuyama M., Wada M., Kataoka H. (2007). AUREOCHROME, a photoreceptor required for photomorphogenesis in stramenopiles. Proc. Natl. Acad. Sci. USA.

[13] Endo H., Okumura Y., Sato Y., Agatsuma Y. (2017). Interactive effects of nutrient availability, temperature, and irradiance on photosynthetic pigments and color of the brown alga *Undaria pinnatifida*. J. Appl. Phycol..

[14] Kim S.H., Kim Y.D., Hwang M.S., Hwang E.K., Yoo H.I. (2021). Temperature ranges for survival and growth of juvenile *Saccharina sculpera* (Laminariales, Phaeophyta) and applications for field cultivation. Algae.

[15] Sato Y., Kozono J., Nishihara G.N., Terada R. (2020). Effect of light and temperature on photosynthesis of a cultivated brown alga, *Saccharina sculpera* (Laminariales), from Japan. Phycologia.

[16] Yoshida T. (1998). Marine Algae of Japan.

[17] Yoshida T., Suzuki M., Yoshinaga K. (2015). Checklist of marine algae of Japan. (revised in 2015). Jpn. J. Phycol..

[18] Shinmura I., Yamanaka K. (1974). Studies on the cultivation of an edible brown alga, *Cladosiphon okamuranus*—I. The season for seeding of zoospore and its growth. Bull. Jpn. Soc. Sci. Fish..

[19] Shinmura I., Yamanaka K. (1974). Studies on the cultivation of an edible brown alga, *Cladosiphon okamuranus*—II. Field culture experiments with a culture-net. Bull. Jpn. Soc. Sci. Fish..

[20] Toma T. (2012). Seaweed and Seagrass in Okinawa.

[21] Toma T., Watanabe M. (2004). Historical development of the mariculture of *Cladosiphon* in Okinawa. Handbook of Algae—Their Diversity and Utilization.

[22] Department of Agriculture, Forestry and Fisheries, Okinawa Prefectural Government (2021). Annual Report of Forest and Forestry of Okinawa.

[23] Sato Y., Nagoe H., Ito M., Konishi T., Fujimura H., Nishihara G.N., Tanaka A. (2021). Final yield of the brown alga *Cladosiphon okamuranus* (Chordariaceae, Phaeophyceae) may depend on nursery quality. Phycol. Res..

[24] Sudo Y., Watanabe M. (2012). *Cladosiphon* *okamuranus*. Handbook of Algae—Their Diversity and Utilization.

[25] Nagoe H., Nishihara G.N., Numata Y., Tozaki K., Maeganeku K., Tanaka A., Sato Y. (2025). When and where should aquaculture nets be deployed in a nursery site to maximize *Cladosiphon okamuranus* growth?. Phycol. Res..

[26] Tozaki K., Nishihara G.N., Kawate A., Konishi T., Sato Y., Ito M., Fujimura H., Tanaka A. (2024). Vegetation variety affected by local environments in a coral reef lagoon. Phycol. Res..

[27] Inomata E., Nagoe H., Tanaka A., Sato Y. (2023). Effects of irradiance and nutrient supplementation on the initial development of erect thalli of different strains of the brown alga *Cladosiphon okamuranus*. Aquac. Sci..

[28] Shinmura I. (1974). Studies on the cultivation of an edible brown alga, *Cladosiphon okamuranus*—III. Development of zoospore from plurilocular sporangium. Bull. Jpn. Soc. Sci. Fish..

[29] Shinmura I. (1975). Studies on the Cultivation of an Edible Brown Alga, *Cladosiphon okamuranus*-IV. Development of Zoospore from Unilocular Sporangium. Nippon Suisan Gakkaishi.

[30] Sudo Y., Yamada S. (2008). Studies of environmental conditions on the macrothalli germination and the growth of *Cladosiphon okamuranus*. Annu. Rep. Okinawa Prefect. Fish. Exp. Stn..

[31] Tanaka A., Takara H., Kamata S., Sato Y., Ueshiro R. (2024). Temperature Influences on Dimorphism of Microthallus in a Brown Alga *Cladosiphon okamuranus*. Cytologia.

[32] Cock J.M., Sterck L., Rouzé P., Scornet D., Allen A.E., Amoutzias G., Anthouard V., Artiguenave F., Aury J.M., Badger J.H. (2010). The *Ectocarpus* genome and the independent evolution of multicellularity in brown algae. Nature.

[33] Nishitsuji K., Arimoto A., Iwai K., Sudo Y., Hisata K., Fujie M., Arakaki N., Kushiro T., Konishi T., Shinzato C. (2016). A draft genome of the brown alga, *Cladosiphon okamuranus*, S-strain: A platform for future studies of ‘mozuku’ biology. DNA Res..

[34] Lüning K., Price J.H., Irvine D.E.G., Farnham W.F. (1980). Control of Algal Life-History by Daylength and Temperature. The Shore Environment.

[35] Nakamura Y., Tatewaki M. (1975). The Life History of Some Species of the Scytosiphonales. Sci. Pap. Inst. Algol. Res. Fac. Sci. Hokkaido Univ..

[36] Navarrete I.A., Kim D.Y., Wilcox C., Reed D.C., Ginsburg D.W., Dutton J.M., Heidelberg J., Raut Y., Wilcox B.H. (2021). Effects of depth-cycling on nutrient uptake and biomass production in the giant kelp *Macrocystis pyrifera*. Renew. Sustain. Energy Rev..

[37] Zhang J., Nagahama T., Ohwaki H., Ishibashi Y., Fujita Y., Yamazaki S. (2004). Analytical approach to the discoloration of edible laver “Nori” in the Ariake Sea. Anal. Sci..

[38] Dan A., Ohno M., Matsuoka M. (2015). Changes of the research and development on the resources of *Undaria* and *Laminaria* in the culture ground of Tokushima coasts. Bull. Tokushima Prefer. Fish. Res. Inst..

[39] Tatewaki M. (1966). Formation of a crustaceous sprophyte with unilocular sporangia in *Scytosiphon lomentaria*. Phycologia.

[40] Shinmura I., Hori T. (1993). *Cladosiphon okamuranus* Tokida. All Illustrated Atlas of the Life History of Algae. Volume 2. Brown and Red Algae.

