# Supplementary material for: Influence of Light Quality on the Initial Development in Edible Brown Alga Cladosiphon okamuranus
Source: Plants (Basel). 2026 Mar 13;15(6):895. doi: 10.3390/plants15060895 (PMC13030692; doi:10.3390/plants15060895)
Supplement: Supplementary file 1 [file plants-15-00895-s001.zip › Table S3.pdf]

Table S3. The influence of environmental factors on the morphological development from discoid thallus to erect sporophyte of *Cladosiphon okamuranus* and *Scytosiphon lomentaria*.

Species	Developmental stage examined	Environmental factors tested	Main findings on sporophyte morphogenesis	Reference
Cladosiphon okamuranus	Discoid thallus<br>→ Erect sporophyte	Temperature (10–30°C)<br>Irradiance (0.5–6 klx)	Optimal sporophyte formation: 15–25°C (optimum ~20°C),1–3 klx<br>Suppressed: ≥25–28°C, 6 klx higher.	[28] Shinmura (1974)
Cladosiphon okamuranus	Discoid thallus<br>→ Assimilatory filaments	Temperature (15–25°C)<br>Salinity (25–35 PSU)	Assimilatory filaments formed: ~20°C, 33–35 PSU<br>Suppressed: Low salinity (<25 PSU) or high temperature (>25°C)	[29]Shinmura (1975)
Cladosiphon okamuranus	Discoid thallus<br>→ Pre-germling formation	Irradiance (~5–80 µmol photons m^{-2} s^{-1})<br>Nutrient enrichment (NE)	Erect thallus formation: 5–20 µmol photons m^{-2} s^{-1}, with NE<br>Supressed: 80 µmol photons m-2 s-1, without NE	[27] Inomata et al. (2023)
Cladosiphon okamuranus	Microthallus dimorphism	Temperature (15–30°C)	Sporophyte-competent morphotypes most abundant: 15–25°C<br>Suppressed: ≥28–30°C.	[31]Tanaka et al. (2024)
Cladosiphon okamuranus	Discoid thallus<br>→ Erect sporophyte	Temperature (10–30°C)<br>Nitrate (0–150 µM)	Optimal sporophyte formation: 15–25 °C (peak ~20°C), 0–50 µM (nitrate)<br>Supressed: <10ºC and ≥30 ºC, ≥ 150 µM (nitrate)	[30]Sudo & Yamada (2008)
Cladosiphon okamuranus	Discoid thallus<br>→Pre-germling formation	Light color (Blue, Red, White)<br>Irradiance (25 or 100 µmol photons m^{-2} s^{-1})<br>Nutrient enrichment	Induced pre-germling: Blue light, regardless of light intensity and nutrients level<br>No effect: Red light	This study
Scytosiphon lomentaria	Microthallus<br>→ Erect sporophyte	Photoperiod (8–16 h light)<br>Temperature (10–20°C)	A permissive temperature range: 10–18°C<br>Suppressed: ≥20°C.<br>#Photoperiod is the primary regulatory factor, while irradiance is not the primary trigger for sporophyte induction.	[34]Lüning (1980)
Scytosiphon lomentaria	Life-history phase transition	Temperature (~5–25°C)<br>Photoperiod	Induce sporophyte formation: Short day length<br>#Quantitative optimal irradiance values were not determined.	[35]Nakamura and Tatewaki (1975)
