# Supplementary material for: Influence of Light Quality on the Initial Development in Edible Brown Alga Cladosiphon okamuranus
Source: Plants (Basel). 2026 Mar 13;15(6):895. doi: 10.3390/plants15060895 (PMC13030692; doi:10.3390/plants15060895)
Supplement: Supplementary file 1 [file plants-15-00895-s001.zip › Table S1.pdf]

Table S1

Results of two-way ANOVA of pre-germling formation rate of *Cladosiphon okamuranus* in sterilized seawater and PESI medium under different light wavelength. N and W indicate nutrient and wavelength, respectively.

		F	df	P
Day 14	Nutrient	57.5954	1	<0.0001
	Wavelength	6.6054	3	0.0006
	N × W	6.6054	3	0.0006
Day 20	Nutrient	328.7719	1	<0.0001
	Wavelength	36.9405	3	<0.0001
	N × W	36.9405	3	<0.0001
Day 27	Nutrient	119.9910	1	<0.0001
	Wavelength	22.9403	3	<0.0001
	N × W	18.6862	3	<0.0001
Day 35	Nutrient	137.3218	1	<0.0001
	Wavelength	37.0323	3	<0.0001
	N × W	24.2318	3	<0.0001
Day 42	Nutrient	100.3561	1	<0.0001
	Wavelength	35.9465	3	<0.0001
	N × W	21.6762	3	<0.0001
