# Supplementary material for: Influence of Light Quality on the Initial Development in Edible Brown Alga Cladosiphon okamuranus
Source: Plants (Basel). 2026 Mar 13;15(6):895. doi: 10.3390/plants15060895 (PMC13030692; doi:10.3390/plants15060895)
Supplement: Supplementary file 1 [file plants-15-00895-s001.zip › Table S2.pdf]

Table S2

Results of two-way ANOVA of pre-germling formation rate of *Cladosiphon okamuranus* in sterilized seawater under different light wavelength at 25 or 100 μmol m^{-2} s^{-1}.

L and W indicate light intensity and wavelength, respectively.

		F	df	P
Day 32	Light intensity	1.0000	1	0.3197
	Wavelength	1.0000	2	0.3715
	L × W	1.0000	2	0.3715
Day 39	Light intensity	4.4472	1	0.0374
	Wavelength	12.4446	2	<0.0001
	L × W	1.3103	2	0.2742
Day 46	Light intensity	3.2025	1	0.0765
	Wavelength	20.3245	2	<0.0001
	L × W	1.0092	2	0.3681
