# Supplementary material for: Influence of Light Quality on the Initial Development in Edible Brown Alga Cladosiphon okamuranus
Source: Plants (Basel). 2026 Mar 13;15(6):895. doi: 10.3390/plants15060895 (PMC13030692; doi:10.3390/plants15060895)
Supplement: Supplementary file 1 [file plants-15-00895-s001.zip › Figure S2.pdf]

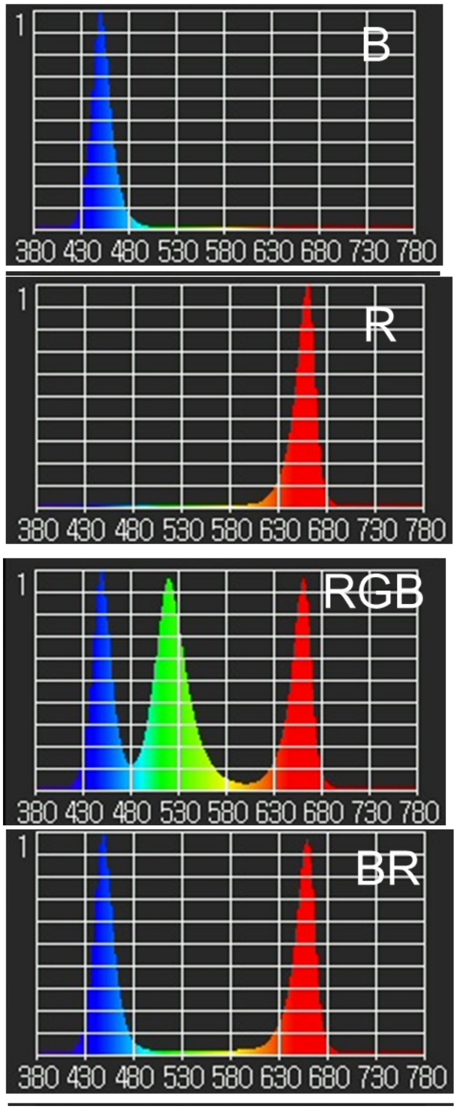

Figure S2. Composition of LED lights set at seven different light wavelength combinations measured by using a light analyzer (LA-105, Nippon Medical & Chemical Instruments, Osaka, Japan).
