# Supplementary figures and images for: Influence of Light Quality on the Initial Development in Edible Brown Alga Cladosiphon okamuranus
Source: Plants (Basel). 2026 Mar 13;15(6):895. doi: 10.3390/plants15060895 (PMC13030692; doi:10.3390/plants15060895)

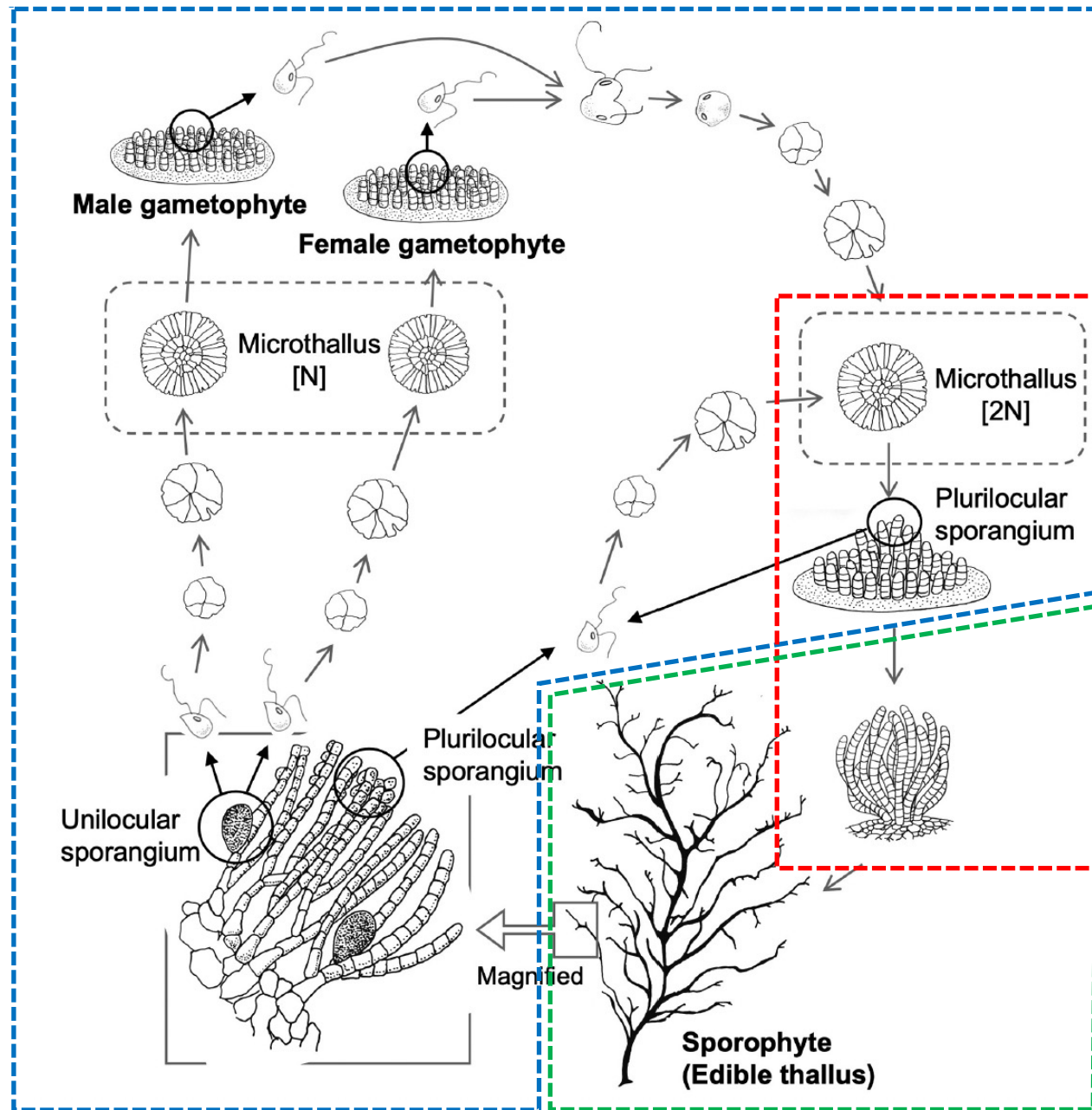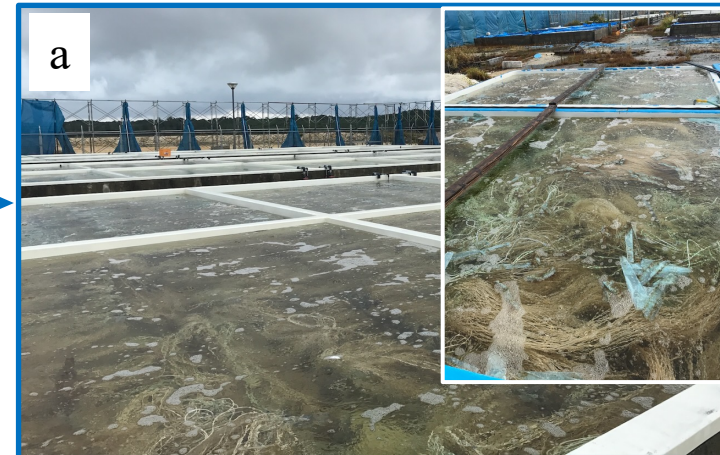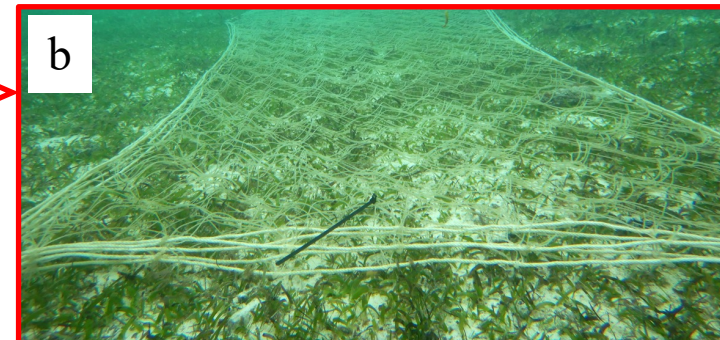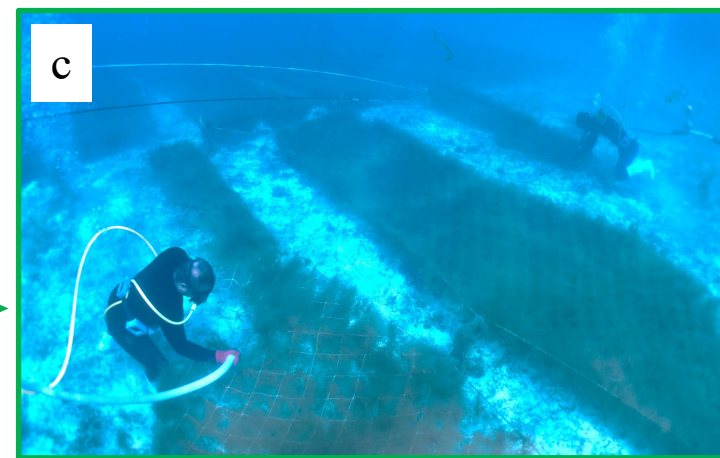

Supplement: Supplementary file 1 [file plants-15-00895-s001.zip › Figure_S1.pdf]
